# Characterization of HTT Inclusion Size, Location, and Timing in the zQ175 Mouse Model of Huntington´s Disease: An *In Vivo* High-Content Imaging Study

**DOI:** 10.1371/journal.pone.0123527

**Published:** 2015-04-10

**Authors:** Nikisha Carty, Nadège Berson, Karsten Tillack, Christina Thiede, Diana Scholz, Karsten Kottig, Yalda Sedaghat, Christina Gabrysiak, George Yohrling, Heinz von der Kammer, Andreas Ebneth, Volker Mack, Ignacio Munoz-Sanjuan, Seung Kwak

**Affiliations:** 1 Evotec AG, Manfred Eigen Campus, Hamburg, Germany; 2 CHDI Management/CHDI Foundation, Princeton, New Jersey, United States of America; University of Florida, UNITED STATES

## Abstract

Huntington’s disease (HD) is an autosomal dominant neurodegenerative disorder caused by a CAG trinucleotide repeat expansion in the *huntingtin* gene. Major pathological hallmarks of HD include inclusions of mutant huntingtin (mHTT) protein, loss of neurons predominantly in the caudate nucleus, and atrophy of multiple brain regions. However, the early sequence of histological events that manifest in region- and cell-specific manner has not been well characterized. Here we use a high-content histological approach to precisely monitor changes in HTT expression and characterize deposition dynamics of mHTT protein inclusion bodies in the recently characterized zQ175 knock-in mouse line. We carried out an automated multi-parameter quantitative analysis of individual cortical and striatal cells in tissue slices from mice aged 2–12 months and confirmed biochemical reports of an age-associated increase in mHTT inclusions in this model. We also found distinct regional and subregional dynamics for inclusion number, size and distribution with subcellular resolution. We used viral-mediated suppression of total HTT in the striatum of zQ175 mice as an example of a therapeutically-relevant but heterogeneously transducing strategy to demonstrate successful application of this platform to quantitatively assess target engagement and outcome on a cellular basis.

## Introduction

Huntington's disease (HD) is an inherited neurodegenerative disorder caused by a CAG repeat encoding a polyglutamine tract within the amino-terminus of the huntingtin (HTT) protein [1]. The disease is clinically characterized by motor, cognitive and psychiatric manifestations [2]. Current understanding of HD is largely based on studies of mutant huntingtin (mHTT)-induced pathogenesis in transgenic mammalian models. A number of HD mouse models that have been extensively characterized [3, 4]. Among these, the knock-in (KI) mouse models bearing an expanded polyglutamine tract within the endogenous murine *HTT* gene locus on chromosome 5q most closely mimic the genetic context of the disorder and express the mutant allele in the endogenous mRNA and protein context [5, 6].

The zQ175 knock-in (KI) mouse line has recently emerged as a particularly useful model to test potential therapeutics since they exhibit extensive behavioral, histopathological, and molecular phenotypes reminiscent of human disease [4, 7]. Amongst the deficits reported, the heterozygote zQ175 mice display age- and region-dependent molecular alterations in genes associated with disease progression, including expression changes in neurotransmitter receptors, BDNF, and DARPP-32, amongst others [8, 9]. zQ175 mice display behavioral deficits, especially in the dark phase of the diurnal cycle at 4.5 months of age, and overt rotarod deficits by 8 months of age, amongst other alterations [8, 9]. Additionally, decreases in striatal Cnr1 and PDE10a mRNA levels are seen in heterozygotes of both sexes by 10 months of age, accompanied by the progressive hyperexcitability of medium spiny neurons, progressive loss of cortical afferent innervation of the striatum, and decreased striatal volumes in heterozygous mice [8].

Given the inverse relationship between age of motoric onset and HTT CAG-repeat length in HD[10], an increase in the CAG length is expected to accelerate and/or magnify HD phenotypes. Here we extend the characterization of the zQ175 KI mice by monitoring HTT expression and inclusion bodies to enable the evaluation of genetic or pharmacological interventions that might affect disease progression [11–13].

High content imaging (HCI) of brain sections is a powerful technique that allows automated quantification of histological endpoints and can be used to detect changes in response to treatments or in the context of genetic perturbations. The Opera platform, formerly used in cellular functional screening assays, enables automated multicolor, multi-parametric fluorescence imaging readouts and, in conjunction with custom adaptation of image analysis algorithms, permits quantitation from thousands of cells in brain sections of large numbers of animals. This allows efficient and thorough analysis of *in vivo* studies following perturbations such as virally-mediated gene delivery, where intra group variability between animals is high with respect to area and cells transduced following intraparenchymal injections.

Here we investigate immunohistological changes in zQ175 heterozygous animals by HCI and describe the alterations in several striatal histological markers [14]. Specifically, we report on the spatial and temporal analysis of mHTT containing EM48-immunoreactive (EM48-ir) species consisting of inclusion bodies, diffuse nuclear signals, and small nuclear puncta across various ages. We also describe the expression of endogenous mouse HTT as detected with the monoclonal antibody MAB2174. These analyses will enable future studies to evaluate the effects of HTT-lowering therapeutic strategies in a quantitative and spatially-informative manner.

## Methods

### Transgenic mice

The zQ175 C57B/L6J knock-in mice, derived from a spontaneous expansion of the CAG copy number in the CAG 140 knock-in mice, were generated at Psychogenics (Tarrytown, NY, USA) and provided for breeding by Jackson Laboratory (Bar Harbor, Maine, USA). Transgenic mice were backcrossed to C57BL/6J to obtain heterozygous zQ175 mice and wild type littermates. Animals were housed in Eurostandard Type II long cages and given access to food and water *ad libitum*. Environmental conditions were maintained at a temperature of 21±1°C, humidity of 55±10% and a 12:12 light:dark cycle with lights on at 7 am and off at 7 pm. During housing, animals were checked daily for health status. All animal work was carried out in accordance with the regulations of the German animal welfare act and the EU legislation (EU directive 2010/63/EU). The protocol was approved by the local ethics committee of the Authority for Health and Consumer Protection of the city and state Hamburg (“*Behörde für Gesundheit und Verbraucherschutz”* BGV, Hamburg) under the file number #V11307/591 00.33. Doxycycline inducible Htt RNAi mice were created by Artemis/Taconic Inc (Cologne, Germany) using their proprietary platform as described previously [15]. The mice were fed doxycycline (625mg/Kg; Harlan Laboratories, Fredrick, MD) in chow feed starting at weaning and sacrificed at 4 months of age.

### AAV production

Plasmids for expression of shRNAs were modified from the Adeno-associated virus (AAV) vector pAAV-6P-SEWB [16]. For expression of shRNAs, transcriptional control units containing the H1 promoter followed by DNA-encoded short hairpin constructs were inserted into pAAV-6P-SEWB to generate AAV vectors with bicistronic expression units. Each viral vector mediated P_Synapsin_-driven EGFP and P_H1_-driven shRNA expression.

shRNA sequences: Two non-allele specific shRNAs targeting murine Htt- mHtt-sh#2 (ggaggacacagtacttcta) and mHtt-sh#4 (AGCTTGTCCAGGTTTATGAA; published previously as sh8.2[17]; control shC004 (cgtgatcttcaccgacaagat),an shRNA without known target sequence in the mouse.

Pseudotyped rAAV2/1+2 particles were produced and purified, as described previously [18]. In brief, HEK293 cells were co-transfected with an AAV vector carrying the transcription units of interest and helper plasmids in equimolar ratios by polyethyleneimine-mediated plasmid transfection. Cells were lysed 48 hours after transfection by three freeze-thaw cycles, and the cell debris was removed by centrifugation. The supernatant containing the viral particles was treated with benzonase, and viral particles were subjected to iodixanol density centrifugation (S6, S7) at 60.000 rpm. Iodixanol was removed, and the viral particles were concentrated in PBS 300 MK (300mM NaCl, 1mM MgCl2, 2.5mM KCl) by filter centrifugation. Remaining rAAV solution was filtered through a Milex GV 0.22 μm pore size. Sterile rAAV particles were stored at 4°C and diluted 1:1 with sterile PB buffer to obtain PBS MK (150 mM NaCl, 0.5mM MgCl2, 1.2 5mM KCl) prior to in vivo application.

### Neonatal AAV application

For rAAV injections, neonate heterozygous zQ175 C57BL/6J mice of both genders were deeply anesthetized by isoflurane inhalation (induction 4%; reduced to 1–2% for surgery at a flow rate of 0.5–1 L/min in pure oxygen). All efforts were made to minimize suffering. Animals were kept on warming pad until fully recovery from anesthesia before placing them back into the mother´s cage. Injected mice were checked daily to ensure the absence of any complications after operation. The rAAV particles encoding shRNA directed against huntingtin were injected into the lateral ventricle of isoflurane anaesthetized newborn (P0) zQ175 C57/BL6 mice KI mice at a concentration of 1E13 vg/ml in a volume of 2 μl, coordinates: 2 mm rostral and 0.7 mm lateral from lambda at a depth of 2 mm measured from the skin. A control group of neonates was injected with rAAV encoding control shRNA directed against turboGFP.

### Histology and immunohistochemistry

Mice were euthanized at the age of 4 months by transcardial perfusion. For perfusions mice were deeply anesthetized by intraperitoneal injection of ketamine/xylazine mixture (120 mg/15 mg per kg in 15 μl/g body weight) using small diameter 27G needles. Before starting the perfusion animals were assessed for the loss of toe pinch reflex and corneal reflex to ensure the correct level of anesthesia was achieved. Mice were transcardially perfused with 30 ml of ice-cold PBS followed by 50 ml of 4% paraformaldehyde using a peristaltic pump. Brain samples were removed from the skull and post-fixed overnight in the same fixative at 4°C, and cryoprotected by incubating in 30% sucrose solutions until saturated. Whole brains were embedded in TissueTek and stored at -80°C. Coronal sections of 25 μm were cut using a cryostat, collected as free floating in 24-well plates, and directly used for staining or stored in a cryoprotection solution (25 mM Na-phosphate buffer pH 7.4, 30% ethylene glycol, 20% glycerol) at -20° C until time of use. The following primary antibodies were used for immunostaining: monoclonal mouse anti-mutant huntingtin (1:100; EM48, Millipore, MAB5374, lot#2135055), monoclonal rat anti-pan huntingtin (1:500; Millipore, MAB2174-100-KL, lot#2290293), monoclonal rabbit anti-DARPP-32 (1:250; clone 19A3, Cell Signaling, #2306, lot#2), polyclonal rabbit anti-NeuN (1:1000; Millipore, ABN78, lot#2140086). All staining were performed with floating sections. Sections were permeabilized in 0.3% Triton X-100/PBS, blocked in 10% normal goat serum/PBS and incubated with the primary antibody diluted in 1% normal goat serum, 0.1% Triton X-100 in PBS at 4°C overnight. Sections were washed three times in PBS for 15 min and incubated in secondary antibody for 2 hours at room temperature. Sections were washed in PBS as described above and mounted using aqueous mounting medium containing DAPI (Fluoroshield, Sigma, F6057) in 24-well glass-bottom plates (Sensoplate, Greiner, #662892) suitable for imaging with the Opera High Content Screening system (PerkinElmer Inc.).

### Image acquisition

Automated image acquisition was conducted using the Opera High Content Screening system and Opera software 2.0.1 (PerkinElmer Inc.) with a 40x water immersion objective (Olympus, NA 1.15, pixel size: 0.32 μm). To reduce fluorescent crosstalk, images were acquired in a multi-exposure experiment with the following filter settings: primary dichroic (405/ 488/561/640 nm) in combination with a 568 nm secondary dichroic for camera 1 and camera 2, 405 nm laser and BP450/50 towards camera 1, 488 nm laser and BP540/75 towards camera 1, 561 nm laser and BP600/40 towards camera 2 and 640 nm laser and BP690/70 towards camera 3. Laser intensity profiles and distortion between the individual cameras were analyzed and corrected for each set of samples using the integrated flatfield correction by reference images and skewcrop algorithms. Multi-field images were acquired covering the region of interest with up to 640 fields per section. Image data was imported into the Columbus image data management and analysis system version 2.4 (PerkinElmer Inc.). Individual fields for each section were selected according to striatal or cortical brain region. Manual segmentation of the striatum was performed by subdividing the region into four quadrants: medial dorsal (md), medial ventral (mv), lateral dorsal (ld) and lateral ventral (lv). The cortex was subdivided into ccx and mcx using reference coordinates (AP 1.0-(-0.1) mm, ML 0–0.5 mm, DV 1.0–2.0 mm and AP 1.0-(-0.1) mm, ML 0–1.3 mm, DV 0–1.5 mm) as guidelines. This information was used during the image processing step to group the readout parameters and assign them to different subregions of the brain.

### Automated image analysis

Image analysis scripts for characterization and quantification of mHTT inclusions were developed using Acapella Studio 3.1 (PerkinElmer Inc.) and the integrated Acapella batch analysis as part of the Columbus system. For all analyses individual cells within tissue sections were identified using the DAPI signal and a general nuclei detection script based on the Acapella “nuclei detection_C” algorithm. Specifically, the algorithm was defined to exclude nuclei with an area smaller than 280px. Before detection of nuclei, noise and background signal was removed from the images by applying a sliding parabola filter (Acapella setting: curvature 5) on the DAPI signal.

Subpopulation of neurons, in particular, MSNs and transduced cells, were identified based on, NeuN-ir, DARPP-32-ir and GFP signal intensities, respectively. Images of NeuN-ir and DARPP-32-ir signals were processed using the Acapella “cytoplasm_detection_C” algorithm to define cytoplasmic and extracellular compartments. Subsequently, the “local” background signal intensity was determined within an extracellular rim region of 3px width and 2px distance from the previously determined cell border. Finally, cells with nuclear signal intensities higher than the mean “local” background by a factor of 1.1, 2.0 or 1.5, were considered DARPP-32, NeuN or GFP positive, respectively.

Characterization and distribution of mHTT inclusions based on EM48-ir detection, was performed by first creating images with reduced background signals using a sliding parabola filter (curvature 20). Next, coordinate positions of pixels with the highest peak intensity values were detected and defined as objects. Once defined, adjacent pixels with intensity values > 2 times the average background intensity (median intensity of whole filtered image) were added to objects in step wise increments. In this manner a new ring of pixels was consecutively added, enlarging the object size until reaching adjacent pixels with intensity values < background intensity. The grown objects were then characterized and sorted based on size and shape. Spots larger than 5px², with a roundness value between 0.5 and 1.2 as well as width to length ratio above 0.4 were defined as mHTT inclusions. Objects with intensity lower than 2 times that of the mean spot intensity of the unfiltered image were excluded from the analysis. In addition to the nuclear region, an extranuclear region was defined as a ring region, 9px in width, outside the nucleus border. While the algorithm used to define this rim may have included an area extracellular to the cell border in some instances, inclusions were always observed to be within cell soma and thus not expected to influence the data analysis. Objects qualifying as mHTT inclusion spots were categorized as nuclear or extranuclear based on the localization of their geometric center. Multiple parameters including the size, density and total number, of nuclear and extranuclear inclusions were quantified and plotted as a ratio per extra-/ nuclear area, per μm^2^, or per DARPP-32 positive cell. In addition to characterizing mHTT inclusion number and size, a second, multi-dimensional, parameter using a texture-based analysis of the nuclear region was evaluated by applying the Acapella “SER-texture” algorithm. The spot-related texture value of this algorithm takes into account both the distribution and fluctuations of the EM48-ir signal intensity within the nuclear region. This unique attribute can be described as the mHTT granularity.

Characterization of endogenous HTT expression was performed using the MAB2174-ir signal. MAB2174-ir intensity within the nuclear and cytoplasm regions of cortical neurons and transduced MSNs were adjusted by subtracting the mean “local” background intensity to exclude non-specific staining. MAB2174-ir intensity was graphed as percent of control.

### Statistical analysis

Statistical analyses were conducted using GraphPad Prism 6.0 software. For all analyses, p-values less than 0.05 were considered statistically significant. Quantitative analyses were performed using one-way ANOVA analyses and Sidak´s post-hoc multiple comparisons test averaging 6–12 sections/animal.

## Results

### High content histology platform

**Fig 1:**
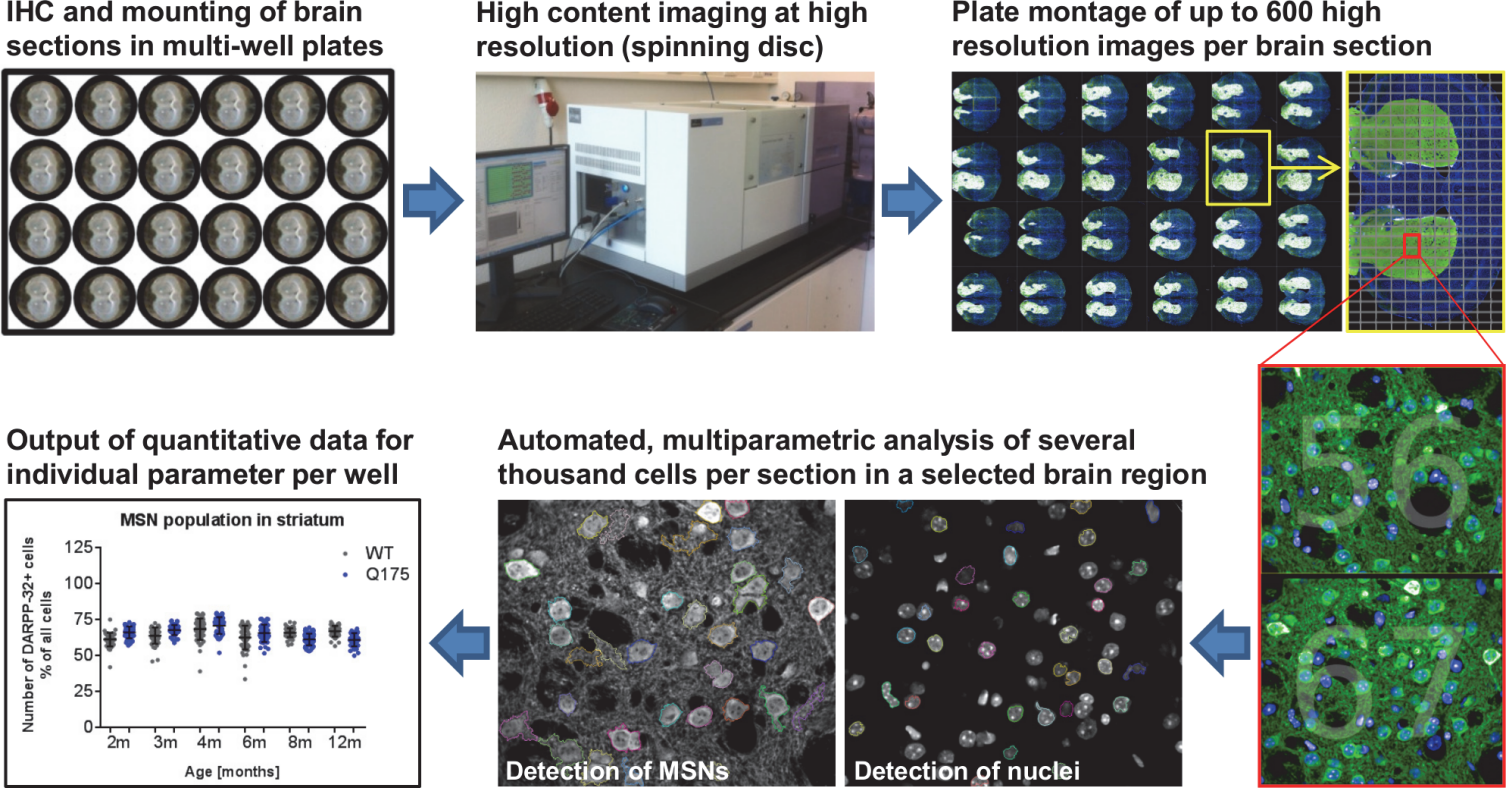
Workflow of high content imaging for *ex vivo* phenotypic characterization. Mouse brain sections fluorescently stained for up to three target proteins were aligned and mounted in glass-bottom multi-well plates suitable for high content imaging. Using an automated imaging setup (Opera, PerkinElmer) up to 600 high resolution confocal images per brain section were acquired. Automated multi-parametric analysis was applied to every single image to generate comprehensive quantitative data sets for each section describing numbers, subcellular structures, morphology and intensities for stained subpopulation of cells.

We developed a streamlined process involving immunohistological staining, automated confocal microscopy, and multiparametric image analysis for fluorescently labeled proteins in rodent brain tissues (Fig 1). Opera High Content Screening system (PerkinElmer Inc.) was adapted for mouse brain multiwells to collect the entire coronal image (~ 600 fields per section). Image segmentation into cortical and striatal subregions enable subcellular analysis of up to three fluorescently-labeled proteins using a proprietary script based on existing analysis software (Acapella, PerkinElmer Inc.), resulting in an average of 50,000 cells per striatum per mouse, and a total of 50 mice to be analyzed within an overall period of 2–3 weeks. Fig 1 describes DARPP32-positive cell count/all cells as one representative of the multi-parametric data sets collected from tissues at the subcellular level.

### Detection of mHTT aggregates

**Fig 2:**
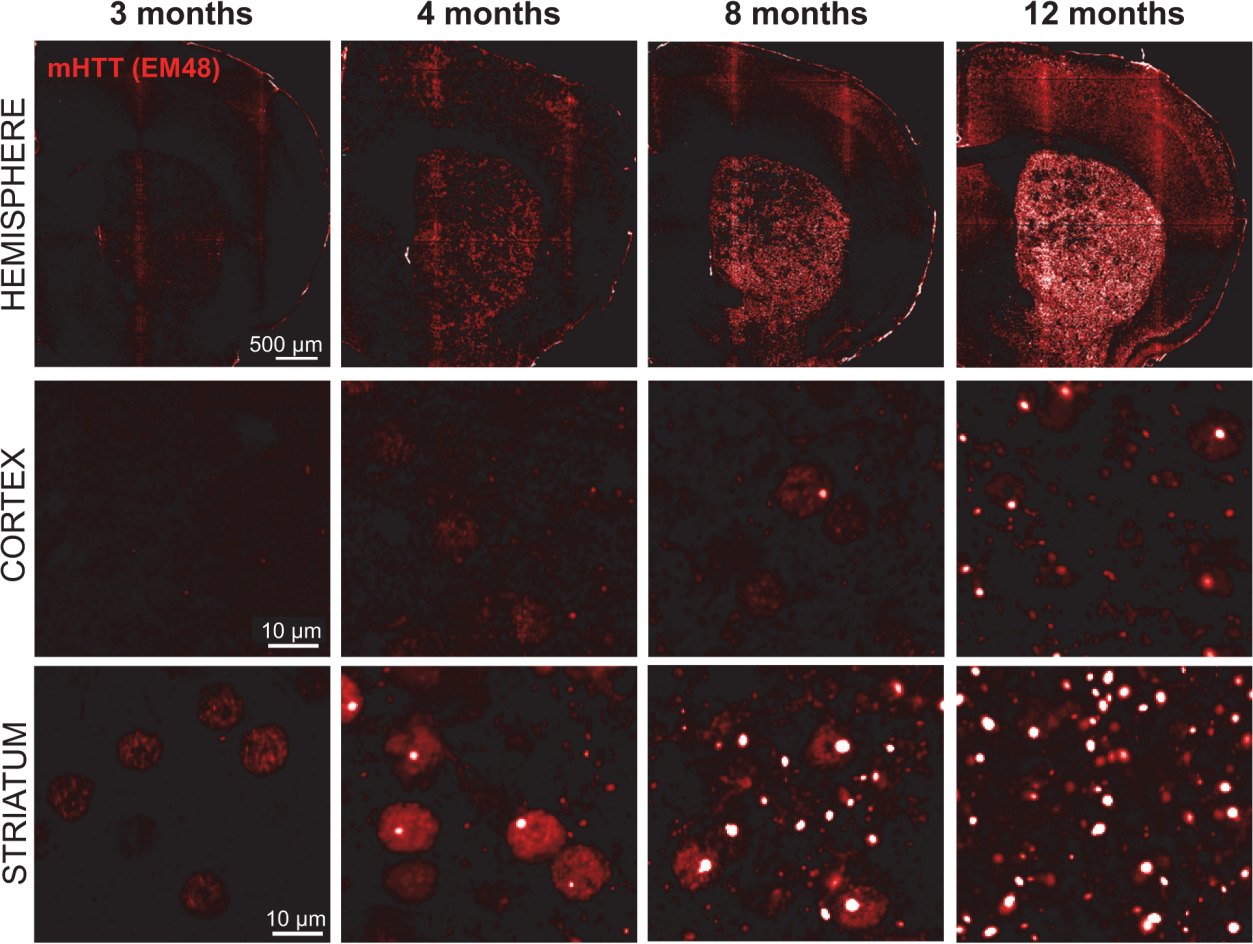
Time course of mHTT inclusions appearance in the striatum and cortex of zQ175 mice. Brain samples from 3–12 months old zQ175 heterozygous mice were stained for mHTT inclusions (bright spots) by EM48-ir and imaged on the Opera high content microscope. A progressive increase in EM48 signal in both striatal and cortical brain regions was clearly visible with age. mHTT inclusions as indicated by EM48-ir puncta appeared earlier and with higher abundance in the striatum as compared to cortex.

In order to establish a time course for mHTT aggregate formation, coronal brains sections (n = 8 mice per age, 6 brain sections per animal) from heterozygous zQ175 mice, 3 to 12 months of age, were immunostained with EM48 antibody. As described for the parent mouse line Hdh140 [6], we identified distinctive patterns of EM48 positive mHTT accumulation that showed a progressive age-dependent increase in size and density in the striatum and cortex (Fig 2). EM48-positive nuclear inclusions, with a distinct punctate staining pattern, were first detected in the striatum at 4 months of age, while the appearance of mHTT nuclear inclusions in the cortex were undetectable until at least 8 months of age (Fig 2). Overall, the age-dependent increase in both number and size of mHTT inclusions was most apparent in the striatum.

In addition to the punctate EM48-ir signal, we also observed a diffuse nuclear EM48-ir signal which, unlike the punctate aggregated protein, first became visible as early as 3 months of age in the striatum but was not detected until 8 months of age in the cortex (Fig 2). Interestingly, both the aggregated and diffuse EM48-ir showed a non-uniform distribution pattern initiating in the striatum, in support of the notion that striatal MSNs are particularly vulnerable to cellular events mediating aggregate formation. Aggregation in other striatal cells (astrocytes, microglia or interneurons) can easily be measured by including other cell-specific markers in the analyses.

### Automated image analysis to characterize mHTT aggregates

**Fig 3:**
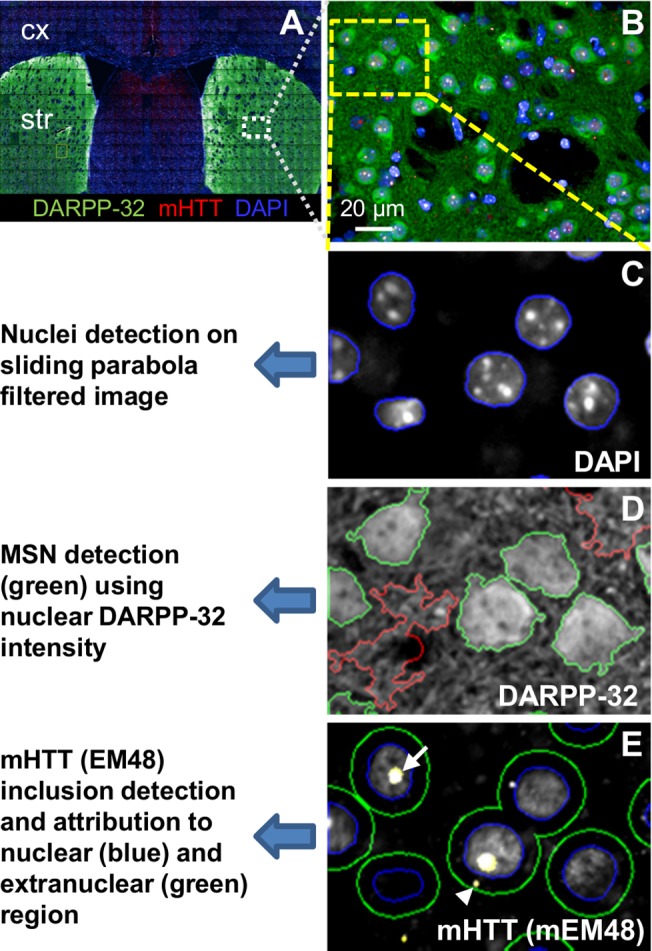
Illustration of inclusion quantification in the striatum of zQ175 mice. Micrographs showing image segmentation strategy for the automated analysis of mHTT inclusion numbers, localization and size in the striatum. (**A**) Coronal brain sections were co-immunostained for detection of DAPRPP-32 and mHTT and image acquisition of striatal and cortical regions was performed with the Opera (PerkinElmer Inc.). (**B**) Multi-field images were acquired using 40x objective lens corresponding to the region of interest for subcellular resolution. (**C**) Area of cell nuclei was determined based on the DAPI signal using a sliding parabola filter for background correction. (**D**) Medium spiny neurons were identified using nuclear intensity of DARPP-32 staining (green). (**E**) An extranuclear region was defined with 10 pixels spacing to the nuclear region. Numbers of mHTT nuclear and extranuclear inclusions in medium spiny neurons (MSNs) were quantified based on EM48 signal.

We used an automated script-based analysis technique to quantify changes observed in EM48-ir inclusions on an individual cell basis in brain tissue sections from zQ175 mice, 2–12 months of age. To achieve a comprehensive analysis of aggregation dynamics in a subpopulation of neurons, brain sections were co-immunolabeled for detection of mHTT and DARPP-32 (a marker for MSNs) and subsequently counterstained with DAPI (Fig 3A). Quantitative image analysis was performed on multiple image fields (40x magnifications) acquired with the Opera High Content Screening system to determine abundance, sub-cellular localization and size of mHTT inclusions in DAPRPP-32 positive MSNs (Fig 3B). A customized automated script analysis using a sliding parabola filtered DAPI image was implemented to identify a nuclear region of individual cells (Fig 3C). The average “local” background intensity of DARPP-32 staining was then determined within a rim region outside the cell boundary. DARPP-32 positive MSNs were identified as having a defined nuclear DARPP-32 intensity value above the local background intensity. Any DARPP-32-ir positive area without a corresponding nuclear region was excluded from the analysis (Fig 3D), and extranuclear rim was defined around the nuclear border. EM48-ir was used to detect and characterize mHTT inclusions within the defined nuclear and extranuclear compartments of DARPP-32 positive MSNs (Fig 3E).

In addition to characterizing the subcellular distribution profile of mHTT, three dynamic features of the EM48-ir signal were also quantified in striatal and cortical regions. These features included number and size of nuclear inclusions (defined as size of > 0.5 μm^2^), as well as density of extranuclear inclusions. We observed that the number and size of discrete nuclear mHTT inclusions followed different kinetics in the striatum compared to the cortex.

**Fig 4:**
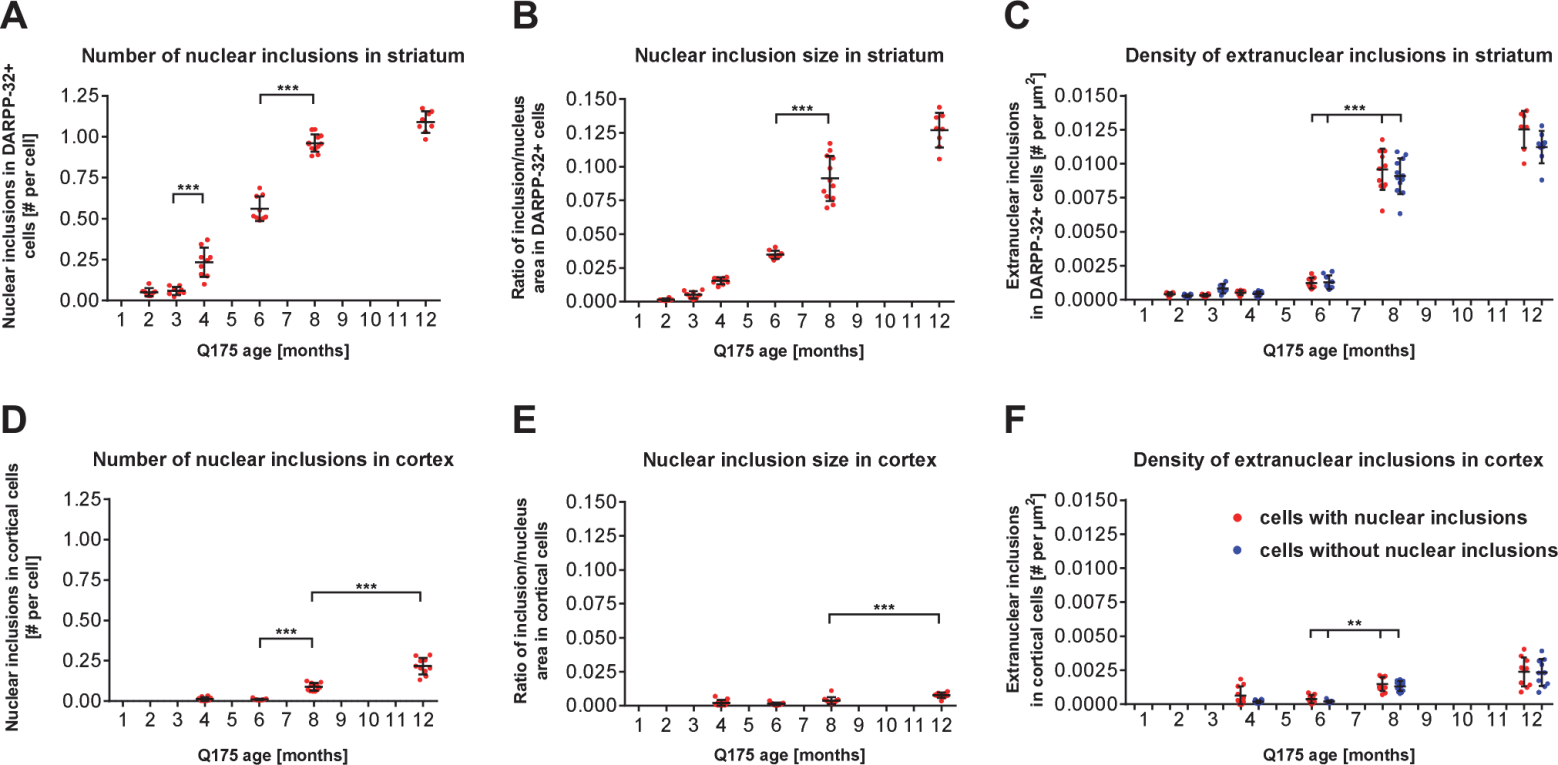
Quantification of nuclear and extranuclear EM48-ir inclusions in the striatum and cortex of zQ175 heterozygous mice. Brain sections of zQ175 heterozygous mice up to 12 months of age were subjected to immunohistochemical staining for DARPP-32 and EM48, followed by the analysis of inclusion number, size and distribution. (**A**) In the striatum the number of nuclear mHtt inclusions are significantly increase between 3 and 4 month of age and between 6 to 8 months, reaching a plateau at 12 month. (**B**) Striatal nuclear inclusions increase steadily in size from 4 to 12 months of age with a significant increase occurring between 6 and 8 months. (**D**) Nuclear mHtt inclusions in the cortex show a delayed kinetics with a significant increase in number observed among 6, 8, and 12 months of age and (**E**) a significant increase in size from 8 to 12 months of age. The number of extranuclear inclusions was normalized to the total area of cells measured and reported as density values. The density of extranuclear inclusions significantly increased between 4 and 8 months of age in the striatum (**C**) and between 6 and 8 months of age in the cortex (**F**). Data are displayed as dot plots with mean +/- SD. Statistical analysis was performed using standard ANOVA and Sidak’s multiple comparisons’ test. For every age an n of 8 animals with 6 sections per animal were used for quantitation; *p<0.05; **p<0.01; ***p<0.001.

Using our script-based image analysis we determined that mean number of nuclear inclusions per DARPP-32 positive cells in the striatum was significantly increased by 4-fold between 3 and 4 months of age (mean values = 0.059 ± 0.026 and 0.24 ± 0.094, respectively; *P* < 0.05). A 2-fold increase in the number of inclusions per cell was observed between 4 and 8 months (mean values = 0.55 ± 0.078 and 0.96 ± 0.058, respectively; *P* < 0.01) (Fig 4A). By 12 months of age this number increased only slightly to 1.1, demonstrating a dramatic reduction in the rate of newly formed aggregates between 8 and 12 months (mean values = 0.96 ± 0.058 and 1.084 ± 0.072, respectively) (Fig 4A). Likewise, an 8-fold increase in the size of striatal nuclear inclusions, calculated as a ratio of inclusion number to nuclear area, was observed between 2 and 8 months followed by a 1.3-fold increase in size from 8 to 12 months of age (*P* < 0.01; Fig 4B). Interestingly, inclusions in the extranuclear compartment showed a slightly altered deposition profile with EM48-ir compared to nuclear inclusions, becoming visible at 6 months and significantly increasing (*P* < 0.001) until reaching a peak density at 8 months of age (Fig 4C). No obvious differences in the density of extranuclear inclusions were observed between cells that did or did not contain mHTT nuclear inclusions (Fig 4C).

In the motor and pre-motor cortex of zQ175 mice, EM48-ir inclusions were initially detected at 6 months. Followed by a significant increase in the inclusion number occurring between 6 and 8 months (*P <* 0.001; Fig 4D) and a significant increase in both number and size between 8 and 12 months of age (*P* < 0.001 respectively; Fig 4D and 4E). The most substantial changes in both the number and size of cortical nuclear mHTT inclusions were detected between 6 and 8 months of age, while the most significant changes in the same parameters were observed at a much earlier time point in the striatum; between 3 and 4 months of age. Furthermore, the average size of mHTT inclusions appeared to be much smaller in the total population of cortical neurons (including all neuronal types) when compared to the size of inclusions in striatal MSNs in all age groups investigated (Fig 4B and 4E).

Our analysis also showed that the density of extranuclear mHTT inclusions in the cortex of zQ175 mice follow a pattern of steady increase starting at 6 months of age and continuing until 12 months, the latest age monitored in this study (Fig 4F). Similar to the nuclear aggregates, the density of extranuclear mHTT inclusions was also found to be higher in the striatum than in the cortex (approximately 5-fold difference was observed at 12 months of age).

### Regional analysis of aggregates appearance

**Fig 5:**
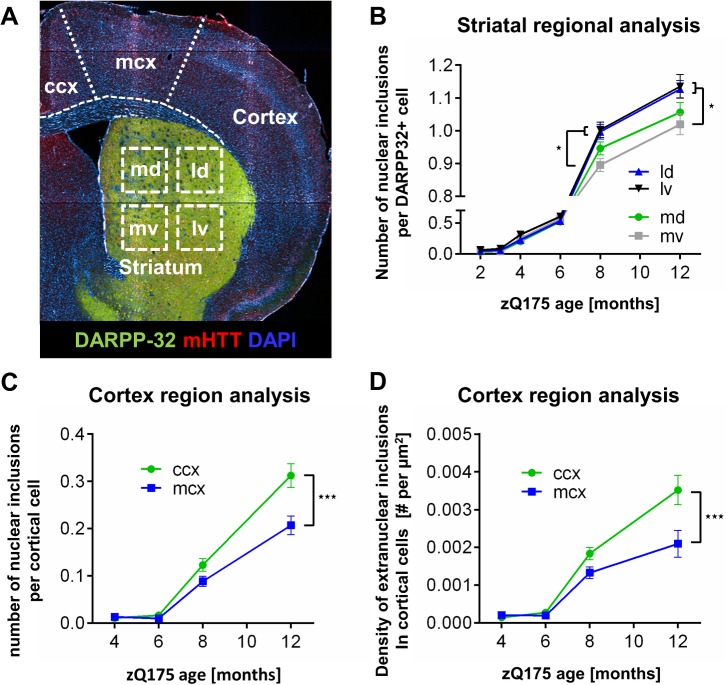
Inclusion appearance in various cortical and striatal regions in zQ175 heterozygous mice. (**A**) Using an automated microscope, whole mouse brain sections were scanned by high resolution multi-image acquisition. Individual images were assigned to distinct areas within the cortex including the cingulate cortex (ccx) and motor cortex (mcx), or within the striatum, including dorsal (d)/ventral (v) and medial (m)/lateral (l) parts to allow region-specific automated multiparametric analysis. (**B**) Region-specific analysis in the striatum of nuclear mHTT inclusions in MSNs. Inclusion number was found to be significantly higher in lateral quadrants (ld and lv) than in medial ventral quadrant at 8 and 12 months old zQ175 mice. (**C**, **D**) Subregion specific analysis in the cortex showing quantification of the number of nuclear (**C**) and extranuclear (**D**) mHTT inclusions in the cingulate and motor cortex over time. A significantly higher number of inclusions were detected in the ccx compared to mcx region in zQ175 heterozygous mice at 12 months of age. Data are displayed as mean +/-SD. Statistical analysis was performed by two-way ANOVA and Sidak’s multiple comparisons’ test. Mean values were calculated for every age and region using an n of 8 animals with 6 sections per animal; *p<0.05; **p<0.01; ***p<0.001.

In order to identify and characterize whether regional patterns of inclusion formation and accumulation exist in this KI line, quantitative analysis was also performed on subregions of the striatum and cortex. In HD pathological analyses, the extent of degeneration and neuronal loss varies along the dorsal/ventral and medio/lateral axes during disease progression [14]. We manually subdivided the striatum into four quadrants: medial dorsal (md), medial ventral (mv), lateral dorsal (ld) and lateral ventral (lv), and the cortex divided into cingulate cortex (ccx) and motor cortex (mcx) (Fig 5A). Image analysis of positive EM48-ir within medium spiny neurons (MSNs), identified by DARPP-32-ir, revealed a significant increase in the number of nuclear inclusions per cell in ld and lv quadrants compared to the mv quadrant at both 8 and 12 months of age (*P* < 0.001; Fig 5B). No differences in inclusion number were observed between medial dorsal and medial ventral quadrants (Fig 5B). To determine the abundance of inclusions and regional relevance to HD in the cortex we characterized nuclear aggregate load in cingulate cortex (ccx) and motor cortex (mcx), respectively. Analysis of both cortical subregions revealed a similar time course of nuclear and extranuclear inclusions formation between 2 to 6 months of age (*P* < 0.001). By 8 months, the cingulate cortex showed a slight elevation in inclusions load compared to motor cortex, which was significantly higher by the age of 12 months (Fig 5C and 5D).

### Nuclear granularity as early phenotypic marker for inclusion formation

As an independent measure of EM48-ir, we also evaluated changes in the diffuse, irregular spatial intensity characteristics of nuclear EM48-ir, defined here as the ‘granularity index’. The texture analysis based method evaluates the pattern of the fluorescence EM48-ir signal in the nuclear region during mHTT inclusion formation. Specifically, the algorithm makes use of the pattern or structure of a fluorescent signal within a specific region of the cell (e.g. the EM48-ir signal in the nuclear region), rather than using the mean intensity, number or position of the signal. Similar detection algorithms have been used for differential analyses of MRI studies in the context of neurodegenerative diseases [19]. Using the Acapella SER (**S**pots, **E**dges and **R**idges) texture analysis features, we examined unique features of mHTT accumulation by using EM48-ir signal intensity structure in the nuclear region, taking into account both the distribution and fluctuations of the EM48-ir signal intensity within the nuclear region.

**Fig 6:**
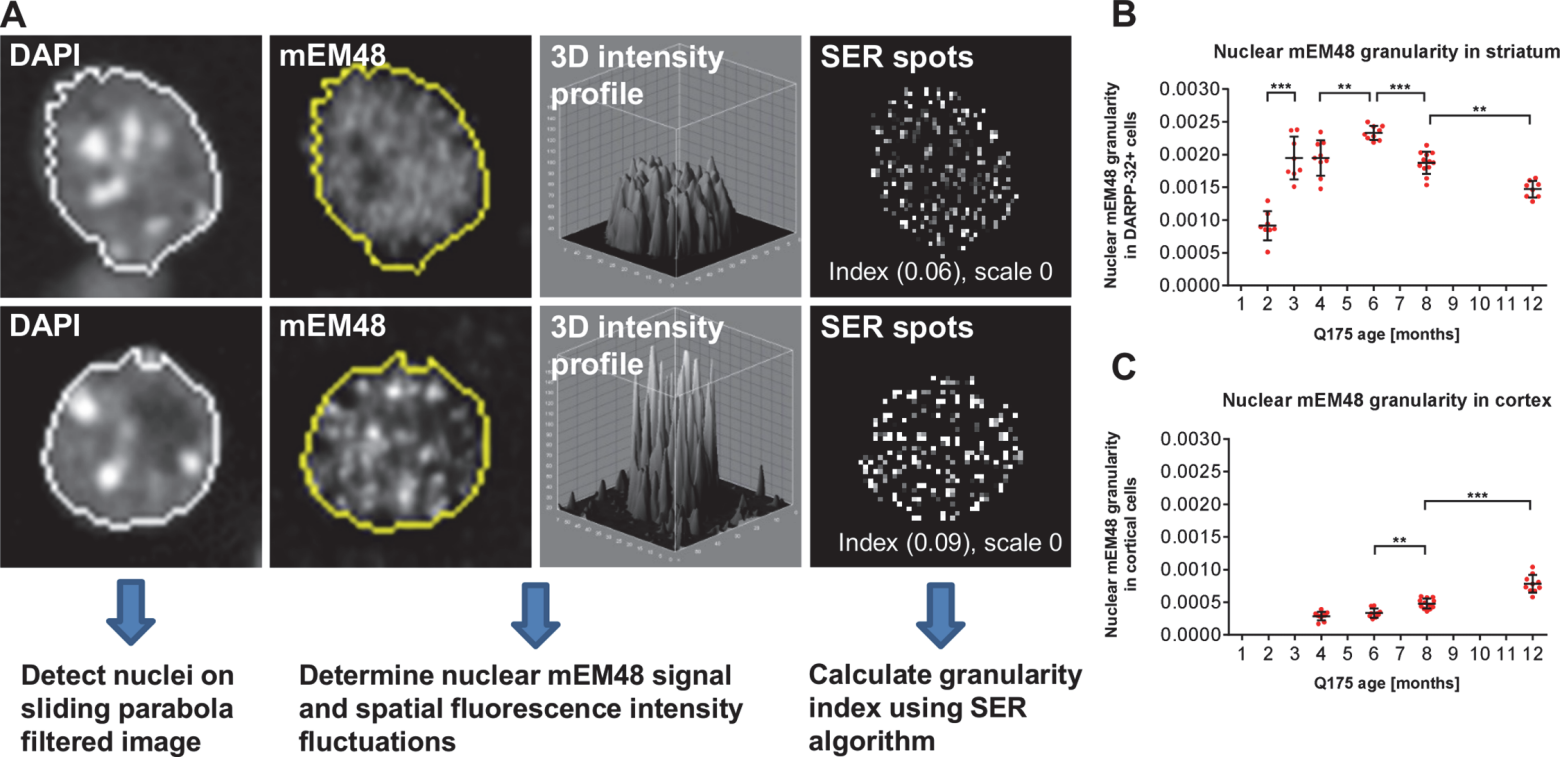
Image texture parameter “granularity” for analysis of early mHTT aggregation. (**A**) Image analysis strategy for early aggregation parameter “granularity”. The area of cell nuclei was detected based on the DAPI signal. The spatial pattern of pixel intensities of nuclear EM48-ir signal was analyzed and a granularity index indicating formation of small EM48-ir inclusion species was calculated using the SER (Spots) texture algorithm (Acapella, PerkinElmer). Brain sections from 2–12 months old zQ175 heterozygote mice were immunolabelled for DARPP-32 and EM48. The “granularity” index in the striatum and cortex was used to detect and monitor early changes in mHTT distribution and signal clustering. (**B**) A significant increase in the nuclear EM48 “granularity” in the striatum was observed from 2 to 6 months of age followed by a significant decrease from 6 to 12 months. (**C**) In the cortex, a significant increase in EM48 granularity was observed from 6 to 12 months. Data are displayed as dot plots with mean +/-SD. Statistical analysis performed using standard ANOVA and Sidak’s multiple comparisons’ test. For every age an n of 8 animals with 6 sections per animal were used for quantitation; *p<0.05; **p<0.01; ***p<0.001.

Overall changes in the granularity of EM48-ir were observed up to four weeks prior to the appearance of discrete inclusions in zQ175 mice. A significant increase in EM48 granularity was observed in striatum between 2 and 3 months of age (*P* < 0.001) and between 4 and 6 months of age (*P* < 0.05; Fig 6B). Interestingly, granularity peaked at 6 months of age and decreased between 6 to 12 months of age (*P* < 0.001; Fig 6B), inversely correlating with the increase in size of EM48-ir inclusions within the nucleus at these ages. In contrast to striatum, a constant (linear) increase in EM48-ir granularity was observed in the cortex of zQ175 mice from 4 to 12 months of age (*P* < 0.001; Fig 6C).

Notably, our analysis indicates that the granularity index is a robust early phenotypic characteristic of mHTT, preceding detection of inclusions, which may provide a novel multi-dimensional parameter to evaluate therapeutic interventions that influence aggregate formation.

### Endogenous HTT detection using MAB2174

**Fig 7:**
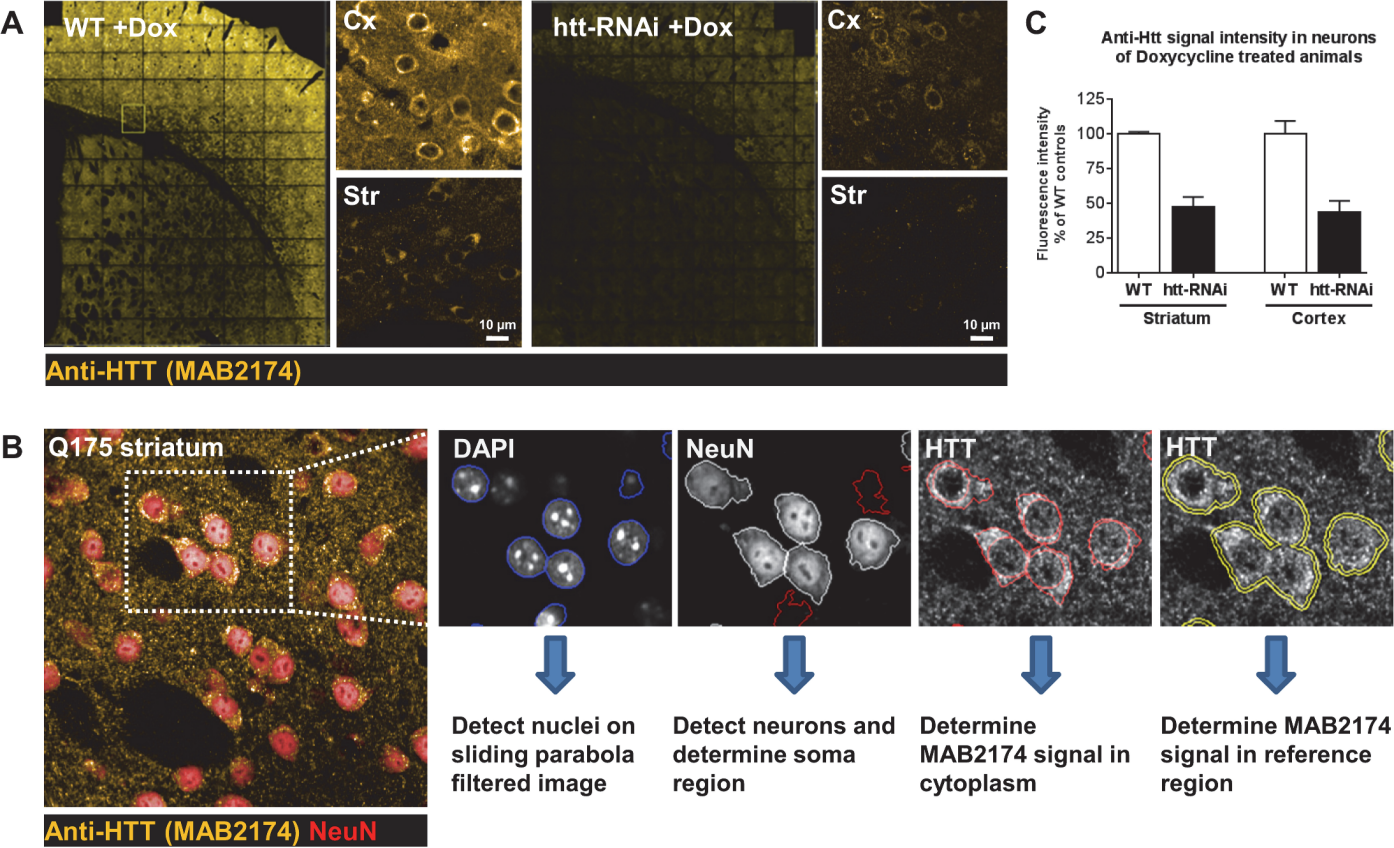
Illustration of pan HTT analysis in zQ175 and HTT conditional knockdown animals. (**A**) Representative images depicting MAB2174-ir in the cortex and striatum of wild type and *Htt* conditional knockdown mice (htt-RNAi). (**B**) Illustration showing the image segmentation for quantifying cytoplasmic endogeneous HTT expression in neurons. Nuclear area was selected based on the DAPI signal. NeuN staining was used to identify neurons and to define the cytoplasmic region. Within this region, HTT levels were quantified by determining mean pixel intensity of MAB2174 antibody staining. Due to variability in staining intensity, MAB2174 signals were corrected for mean fluorescent intensities of a reference region around the cytoplasmic region. (**C**) Quantification of MAB2174 pan HTT staining in the cortex and striatum of wild type and htt-RNAi mice. In both brain regions, MAB2174-ir was below 50% as compared to wild type controls. Data are displayed as bar graphs with mean +/-SD. Mean values were calculated from 1 (striatum) or 2 (cortex) animals with an n of 3 sections per animal.

To characterize HTT distribution and subcellular localization in the Q175 KI mouse line and to evaluate the efficiency of potential therapeutics on HTT protein expression in mice, we evaluated the ability of an anti-HTT antibody, MAB2174, to specifically detect endogenous mouse HTT. To confirm the specificity of MAB2174-ir signal, brain samples from WT and transgenic mice engineered to express anti-HTT shRNA induced by doxycycline-dependent transactivation (HTT-RNAi mice) were co-immunostained with anti-HTT MAB2174 and anti-NeuN antibodies. A comparison of the relative expression levels of HTT in cortical and striatal neurons was performed by quantifying the MAB2174-ir pixel intensity using automated multi-parametric script analysis of multiple high resolution (40x magnification) images (Fig 7A). The script based automated image analysis used DAPI staining and NeuN-ir signals to define the nuclear and cytoplasmic regions, respectively, subsequently allowing for a precise detection and quantification of cytoplasmic MAB2174-ir pixel intensity (Fig 7A and 7B). We observed that the cytoplasmic MAB2174-ir intensity was substantially lower in striatal and cortical neurons of brain sections from doxycycline-treated HTT-RNAi mice as compared to doxycycline-treated WT mice, suggesting specific detection of HTT protein is achieved with this antibody (Fig 7B). A quantitative data analysis indicated a reduction of more than 50% anti-HTT signal in both cortex and striatum of HTT-RNAi as compared to WT mice at 9 weeks of age (Fig 7C). These data are in agreement with previous reports demonstrating that silencing HTT with RNAi in mice results in a significant reduction in HTT mRNA and protein expression [20–22].

### Proof-of-concept: modulation of HTT levels and aggregates

**Fig 8:**
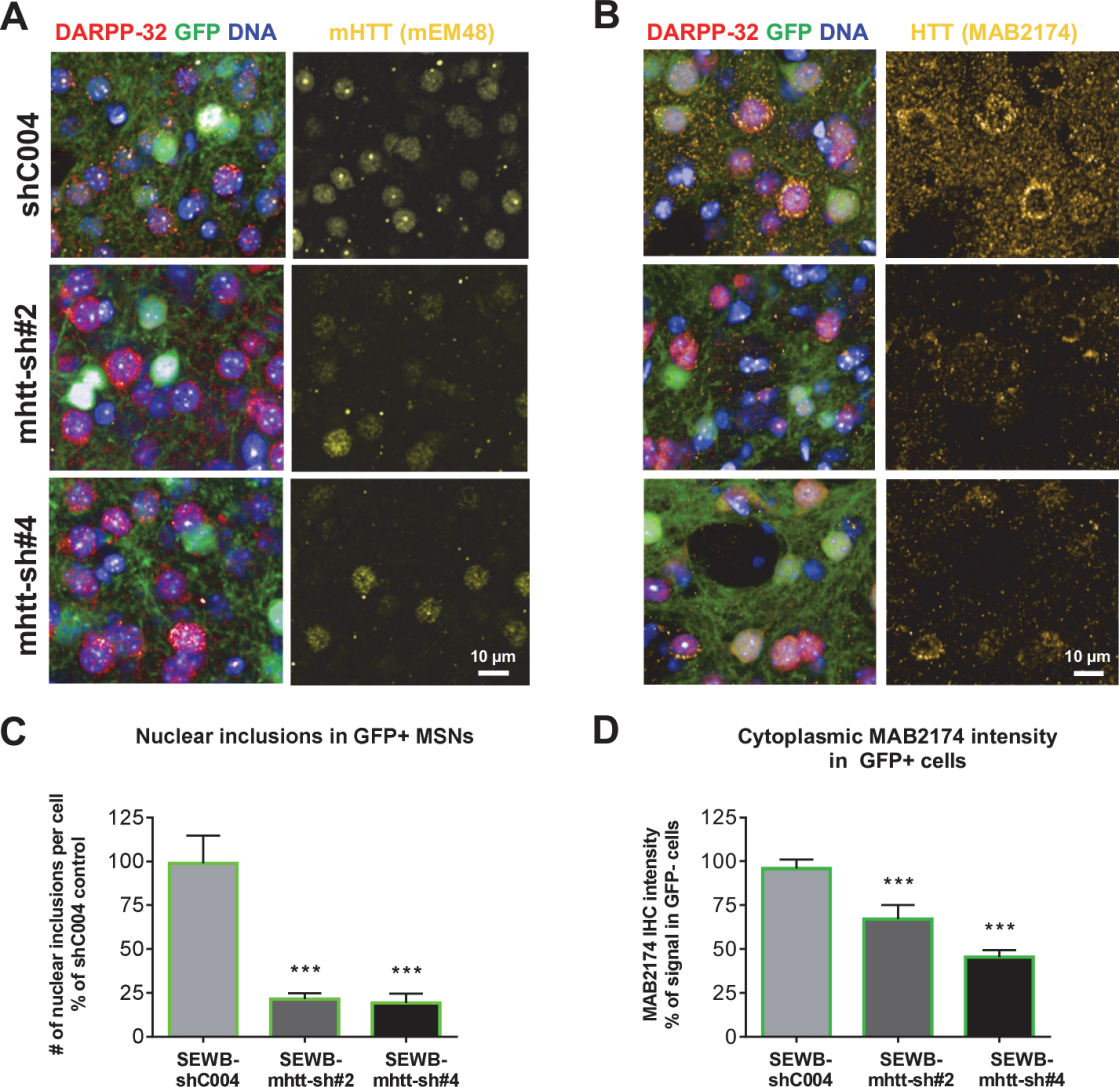
Modulation of HTT levels, as evidenced by MAB2174 and EM48 immunoreactivity in the striatum of zQ175 heterozygous mice by AAV2 viruses expressing HTT-targeting shRNAs. AAV2 viruses encoding GFP and shRNAs directed against HTT were injected in the right ventricle of neonate zQ175 heterozygous mice. At 4 months of age, mice were euthanized and analysed for HTT inclusions and HTT cytoplasmic levels. Representative images showing DARPP-32, mHTT IHC staining (**A**) or DARPP-32, HTT (MAB2174) IHC staining (**B**) in the GFP positive striatal region transduced with AAV2 encoding shRNA against HTT (mHtt-sh#2 and mHtt-sh#4) or non-target control shRNAs (shC004). Quantitative analysis of mHTT inclusions (**C**) and MAB2174-ir intensity (**D**) in GFP positive cells of the striatum: mHtt targeting shRNA led to a significant decrease in the number of nuclear HTT EM48-ir inclusions and MAB2174-ir HTT cytoplasmic levels in comparison to the control AAV/shC004. Data are displayed as bar graphs with mean +/-SD. Statistical analysis was performed using standard ANOVA and Sidak’s multiple comparisons’ test. For every group an n of 4 animals with 3 sections per animal were used for quantitation; p<0.05; **p<0.01; ***p<0.001.

Many current therapeutic programs aimed at lowering HTT use agents such as antisense oligonucleotides (ASO), small interfering RNAs (siRNAs) or microRNAs, which all suffer from limited bio-distribution [23, 24]. To illustrate the practicality and usefulness of our HCI platform as a powerful tool to quantitatively evaluate gene silencing efficiency and related effects on signaling proteins, we administered recombinant adeno-associated virus (AAV) vectors harboring shRNAs targeting *Htt* mRNA to the brains of zQ175 heterozygous mice. Three shRNA-AAVs, shC004 (control shRNA not targeting any known mouse mRNA, *mHtt*-sh#2 and *mHtt*-sh#4 *mHtt* targeting shRNAs), were injected in the ventricles of zQ175 neonates to decrease HTT expression early in postnatal development. All AAV constructs contained a GFP reporter for convenient detection of transduced cells. We initially evaluated the effects of virally-transduced shRNA expression histologically for GFAP and Iba1 immunoreactivity two months following neonatal injection. Neither *mHtt*-sh#2 nor *mHtt*-sh#4 RNAi construct elicited appreciable inflammatory response in this paradigm (data not shown). At 4 months of age, additional mice were euthanized and brains were prepared for IHC. EM48/DARPP-32 co-staining was performed along with DAPI counterstaining. Co-staining with anti-HTT (MAB2174-ir) and anti-NeuN were performed on consecutive brain sections (Fig 8A). We focused on the analysis of the striatum to demonstrate reduction of nuclear EM48-ir inclusions along with decreased HTT immunoreactivity (MAB2174-ir) in *Htt*-shRNA expressing cells. zQ175 mice that received either *mHtt*-sh#2 or *mHtt*-sh#4 AAV intra-striatal injections showed more than 75% reduction in nuclear inclusion count, 78.3% +/-8.8 SD; 80.5% +/-13.2 SD, (Fig 8C) and 33.3% +/-14.1 SD; 54.5% +/-.6 SD reduction in cytoplasmic MAB2174-ir relative to the non-target shRNA mice (Fig 8D). Taken together, these data clearly demonstrate the capacity of our high content histology platform as an unbiased method to investigate the effects of potential therapeutic interventions in the zQ175 HD mouse model by monitoring multi-parameter dynamics of HTT expression and inclusions in the context of subcellular resolution.

## Discussion

Therapies that aim to lower HTT gene expression are currently among the most promising strategies to treat this monogenetic neurodegenerative disease. However, the front runners of this approach—which consist of antisense oligonucleotides, siRNAs, virally mediated RNAi, and DNA-targeting agents—are hampered with delivery and bio-distribution limitations that require local or regional assessment of target engagement[13]. To support preclinical activities in this research area we have developed a suite of algorithms to sensitively measure histological features of HTT reduction and phenotypic outcome at a cellular level. Using EM48-ir as one example of a histological phenotype that can be measured as a consequence of mHTT expression, we used parameters such as inclusion number, size, and intracellular localization on a cellular basis. Our results indicate that the onset of histological dysfunction is accelerated in the heterozygous zQ175 KI model, with EM48-ir observed as early as 3 months of age in the form of diffuse nuclear immunoreactivity, which precedes the earliest age when similar features are first reported in Q111 KI mice by 4 months [12], and even precedes onset of the parental KI Q140 homozygous mice at 2–4 months [6]. Nuclear EM48-ir inclusions are detected after 3 months, are readily quantitated by 4 months, and dynamically increase up to 8 months in the striatum. The timing of this phenotype provides a framework to design HTT suppression studies that either block formation or enhance clearance of protein aggregates. For instance, suppression of HTT in mice that is initiated by 2 months of age should be an effective study plan to evaluate HTT-aggregation blockers, whereas we would propose the use of 6 month old Q175 heterozygous mice to query mechanisms associated with macro-autophagic clearance; at this age macroaggregates have formed and are stable in number, which may help to draw conclusions regarding the applied perturbation on autophagic clearance.

Our analysis revealed that striatal extranuclear EM48-ir inclusions form in a similar timeline to nuclear inclusions, followed two months later by the emergence of cortical inclusions. In both brain regions the emergence of extranuclear inclusions was independent of the presence of nuclear inclusions within the same cell, suggesting that the two processes may be mediated by independent molecular mechanisms. Our interpretation is limited by the histochemical detection of available epitope, such that entities are identified as inclusions when in fact they may exist in various biochemical forms. Therefore relationship among inclusion size, location, and timing reported here may reflect a summation of differing dynamics for different species.

Exploring the idea that irregularity in diffuse nuclear EM48-ir may be related to misfolded oligomers, we refined a texture algorithm to quantitate changes in the appearance and accumulation of this signal. Expressed as a granularity score, we find such signal to be dynamic, appearing as early as 2 months and peaking at 6 months. This signal decreases thereafter, coinciding with the deceleration of increased inclusion formation and size. Together these data are consistent with the notion that inclusions form by sequestration of smaller granular entities [25].

The histopathology in the HD brain has been reported to progress in a gradient along posteroanterior, dorsoventral, and mediolateral axes [14]. The HCI system enables segregation and quantification of regional and subregional effects in the mouse brain. We performed the subregional analysis of cortex and find aggregate formation most robustly in the cingulate cortex of our KI mouse model. However, we failed to detect progressive changes in EM48-ir inclusions as a function of striatal subregion, with the only difference observed being a slight effect in the lateral/medial axis. Perhaps a more relevant subregional analysis may be required to identify striosome versus matrix in these mice, in the nucleus accumbens or globus pallidus regions, as these subdivisions of the basal ganglia have a distinct neuropathological trajectory [14, 26].

We have immunohistochemically characterized a commercially available rat monoclonal antibody, MAB2174, that detects mouse HTT; this antibody was raised against human HTT (residues 549–679aa) and has previously been used in rats and monkeys [27, 28], and it performed well here on mouse brain sections. Notable variance across vendor’s batches was observed, compelling us to stock a single large production run of 100 vials (Lot 2290293). Using this master stock we have been able to evaluate total HTT protein distribution in heterozygous KI mice, evaluate pan-suppression of total HTT by RNAi, and are currently testing mHTT-specific suppression agents for specificity. The MAB2174 is purported to perform well in nonhuman primate sections as well, and this reagent should be useful in various future studies.

Preclinical studies of virally-mediated gene suppression have demonstrated benefit in several HD models [17, 21, 22] but cell-cell variability in viral transduction must be taken into account. The ability to measure HTT proteins *in situ* concordant with additional histological phenotypes will be useful to investigate the relationship between target engagement and outcomes. As an example here, we demonstrate that EM48-ir inclusions and total HTT-ir signal are reduced following AAV-mediated transduction of neonates. We noted specific changes associated with HTT protein, encouraging us to use this platform following adult transduction of HTT suppression agents, where transduced and non-transduced cells in the same and adjacent brain areas can be evaluated simultaneously.

In summary, the current study demonstrates the use of a high-throughput imaging system that, coupled with robust programming and appropriate tissue handling, permits quantitative analysis of histological preparations. Gene therapy strategies for neurodegenerative diseases that require cellular or histological analysis for target engagement and outcome measures may be particularly suited for this platform since the high throughput and cell-counts permit sensitive detection of biological effects.
